# Dynamic Schiff Base Chitosan–Lignin Bioplastics with Thermally Assisted Repair and a Reprocessable Network

**DOI:** 10.3390/ma19194064

**Published:** 2026-09-23

**Authors:** Rysgul Tuleyeva, Nargiz Gizatullina, Madina Mussalimova, Zhanserik Shynykul, Gaukhar Toleutay

**Affiliations:** 1 Department of Chemical and Biochemical Engineering, Geology and Oil-Gas Business Institute Named After K. Turyssov, Satbayev University, 22 Satbayev Str., Almaty 050043, Kazakhstan; rysgultuleyeva@gmail.com (R.T.); gizatullinanargiz.nz@gmail.com (N.G.); mussalimova.m@stud.satbayev.university (M.M.); 2 Department of Chemistry and Technology of Organic Substances, Natural Compounds and Polymers, Faculty of Chemistry and Chemical Technology, Al-Farabi Kazakh National University, 71 Al-Farabi Ave., Almaty 050040, Kazakhstan; 3 Department of Chemistry, University of Tennessee, Knoxville, TN 37996, USA

**Keywords:** chitosan, dialdehyde lignin, bioplastics, bio-based films, Schiff base reaction, thermally assisted repair, reprocessable polymers, sustainable materials

## Abstract

The development of renewable polymer networks combining mechanical performance with repairability and reprocessability is important for advancing sustainable materials. In this study, chitosan-dialdehyde lignin (Ch-DAL) bioplastic films were fabricated through dynamic Schiff base cross-linking between tartaric acid-protonated chitosan and periodate-oxidized lignin, with glycerol as a plasticizer. Films with Ch:DAL ratios of 1:1, 2:1, and 1:2 were characterized by Fourier-transform infrared (FTIR) spectroscopy, tensile testing, qualitative solvent-resistance assessment, a thermally assisted repair test, and two successive thermo-mechanical reprocessing cycles. FTIR analysis was consistent with the possible formation of imine (C=N) linkages and the contribution of hydrogen-bonding interactions, although aldehyde content, degree of conversion, and cross-link density were not quantitatively determined. The mechanical properties were composition-dependent, with the 1:1 formulation exhibiting the highest tensile strength (8.85 ± 0.09 MPa) and Young’s modulus (118.7 ± 0.7 MPa), whereas the 2:1 formulation exhibited the highest elongation at break (238 ± 0.1%). Thermogravimetric analysis (TGA) and derivative thermogravimetry (DTG) showed distinct thermal-degradation profiles for alkali lignin, DAL, and the 1:1 Ch–DAL film, with residual masses of approximately 17%, 57%, and 33% at 600 °C, respectively. Qualitative assessment showed that the films retained structural integrity in water, 1 M sodium hydroxide (NaOH), and the investigated organic solvents but underwent visible fragmentation in 1 M hydrochloric acid (HCl). Following hot pressing of the cut 1:1 film at 50 °C, macroscopic continuity was re-established and tensile strength increased to 12.9 ± 0.11 MPa; however, elongation at break decreased from 19.0 ± 0.1% to 4.3 ± 0.05%, indicating a mechanically distinct repaired state rather than recovery of the original properties. Fragmented films could also be remolded through two successive thermo-mechanical reprocessing cycles, although tensile strength decreased substantially after the first cycle and recovered only partially during the second cycle. These findings demonstrate composition-dependent mechanical behavior, thermally assisted repair, and remolding capability in Ch-DAL films, while further quantitative characterization, durability testing, and environmental assessment are required before claims regarding long-term performance or sustainability can be established.

## 1. Introduction

The increasing accumulation of petroleum-derived plastic waste has intensified efforts to develop sustainable materials from renewable feedstocks. Conventional plastics combine high durability with resistance to environmental degradation, properties that are advantageous during use but contribute to their persistence after disposal. Biopolymers offer an alternative platform because they can be obtained from renewable resources and engineered to provide useful mechanical and functional properties while reducing dependence on fossil-derived materials [1,2,3,4,5,6]. Among these materials, polysaccharides and lignin are particularly attractive because of their abundance, chemical functionality, and potential for constructing biodegradable polymer networks.

Chitosan is a partially deacetylated derivative of chitin that has been widely investigated for films, coatings, and other biobased materials. Its polymer backbone contains abundant amino and hydroxyl groups that enable hydrogen bonding and provide reactive sites for chemical modification [7,8,9]. These characteristics, together with its biocompatibility and film-forming ability, make chitosan an attractive matrix for sustainable polymer systems. However, unmodified chitosan films commonly exhibit brittleness and high hydrophilicity, which can limit their mechanical performance and stability under demanding conditions. Consequently, strategies that reinforce the chitosan network while retaining its renewable character are required to expand its practical applicability.

One approach to overcoming these limitations is to introduce cross-links between chitosan chains using multifunctional biobased components. Cross-linking can restrict excessive polymer-chain mobility, improve network cohesion, and modify the mechanical and chemical properties of the resulting material [10,11]. Permanent covalent cross-links can provide substantial reinforcement but may limit subsequent reshaping and end-of-life processing [12]. Dynamic covalent chemistry provides a different strategy by introducing bonds that retain covalent network integrity while remaining capable of reversible exchange under appropriate conditions [13,14]. Such networks are therefore of particular interest for designing biobased materials that combine mechanical stability with repairability and reprocessability.

In this context, reprocessability, recyclability, and closed-loop recycling describe different levels of material recovery and should not be used interchangeably. Reprocessability refers to the ability of a polymer network to be reshaped or remolded through bond exchange without necessarily recovering its original constituents. Recyclability is a broader concept that may include mechanical or chemical conversion of a used material into a useful subsequent product. Closed-loop recycling more specifically requires recovery of the constituent monomers or precursors, or production of an equivalent material that can re-enter the same manufacturing cycle. Consequently, the ability to consolidate fragmented material by hot pressing demonstrates thermo-mechanical reprocessability but does not, by itself, establish closed-loop recyclability [15,16,17,18].

Lignin represents a promising renewable building block for constructing these networks. As an abundant aromatic component of lignocellulosic biomass, lignin contains phenolic and aliphatic hydroxyl groups that permit chemical functionalization, while its rigid aromatic framework can contribute to mechanical and thermal performance [12,13]. However, the heterogeneous native structure of lignin can restrict its controlled interaction with other polymers. Periodate oxidation provides a route for introducing aldehyde functionalities into lignin, producing dialdehyde lignin (DAL) with enhanced reactivity toward amino-containing polymers [14]. The resulting aldehyde groups can react with the primary amino groups of chitosan to form imine linkages through Schiff base chemistry, thereby integrating lignin into a dynamically cross-linked polymer network.

Previous studies have established several components of this material-design strategy. Chitosan has been processed into films and chemically modified through Schiff base formation to alter its structural, physicochemical, and functional properties [7,8,9,10,11]. Separately, lignin has been used as a renewable reinforcing or binding component, while chemical modification, including periodate oxidation, has been investigated to increase its reactivity and facilitate its incorporation into biobased polymer systems [6,12,13,14]. Research on dynamic imine-containing polymers and polysaccharide networks has further demonstrated that reversible covalent bonds can enable network rearrangement, repair under an external stimulus, and thermo-mechanical reprocessing [15,16,17,18,19,20,21]. Collectively, these studies establish the potential of chitosan, functionalized lignin, and dynamic imine chemistry; however, the preparation–structure–property relationships of cast chitosan–dialdehyde lignin films remain insufficiently defined. In particular, limited information is available on how the Ch:DAL ratio simultaneously influences film formation, mechanical strength and extensibility, visually assessed solvent resistance, thermally assisted repair, and mechanical-property retention during repeated reprocessing.

Schiff base chemistry is particularly attractive because imine bonds can undergo reversible formation and exchange, providing a molecular mechanism for network rearrangement after damage or processing [15,16,17,18,19,20,21]. In chitosan–DAL systems, these dynamic covalent interactions can provide network connectivity while potentially allowing the material to reorganize during thermally assisted repair or reprocessing. This behavior distinguishes dynamic networks from conventional permanently cross-linked thermosets, which generally cannot be readily reshaped after curing. The combination of a polysaccharide matrix with chemically functionalized lignin therefore offers a route toward renewable adaptive materials in which network reinforcement and dynamic behavior originate from complementary functionalities of the two biopolymers.

The properties of such networks are determined not only by dynamic covalent bonds but also by noncovalent interactions and molecular mobility. Hydrogen bonding among chitosan, DAL, and other hydroxyl-containing components can contribute substantially to intermolecular cohesion and influence stiffness, strength, and environmental stability. At the same time, excessive intermolecular association can restrict chain mobility and produce brittle materials. Glycerol can address this limitation by acting as a plasticizer that disrupts some polymer–polymer interactions, increases free volume, and facilitates segmental motion [22,23]. The final material behavior therefore depends on the balance among Schiff base cross-linking, hydrogen bonding, plasticization, and composition, making the chitosan-to-DAL ratio a key parameter for controlling network architecture and macroscopic performance.

Accordingly, this study investigates biobased films prepared from tartaric acid-protonated chitosan and periodate-oxidized DAL, with glycerol incorporated as a plasticizer. The effects of composition and the Ch:DAL ratio on film formation, intermolecular interactions, mechanical properties, qualitative solvent resistance, thermally assisted repair, and reprocessability were evaluated. Particular attention was given to linking the chemical structure and dynamic interactions within the network to the resulting material performance. The novelty of this study lies in combining periodate-oxidized lignin as a multifunctional macromolecular cross-linking component with chitosan and systematically varying the Ch:DAL ratio to determine its relationship with mechanical behavior and adaptive processing responses. Unlike studies addressing these properties individually, the present work compares composition-dependent mechanical performance, qualitative solvent resistance, thermally assisted repair after complete cutting, and property evolution over two successive thermo-mechanical reprocessing cycles within the same Ch-DAL film system. By integrating renewable chitosan and lignin through dynamic Schiff base chemistry, this work aims to establish a tunable biobased material platform that combines mechanical integrity with thermally assisted repair and reprocessing capabilities, without implying that closed-loop recycling has been demonstrated.

## 2. Materials and Methods

### 2.1. Materials

Alkali lignin (product No. 471003; CAS No. 8068-05-1; average molecular weight, Mw ≈ 10,000 g mol^−1^; sulfur content, 4%), sodium periodate (NaIO_4_, ≥99.8%; CAS No. 7790-28-5), chitosan (product No. 448877; molecular weight: 200,000; CAS No. 9012-76-4; average molecular weight, Mw ≈ 200,000 g mol^−1^; degree of deacetylation, ≥75%), L-(+)-tartaric acid (≥99.5%; CAS No. 87-69-4), glycerol (99.5%; CAS No. 56-81-5), and ethanol (99.5%; CAS No. 64-17-5) were purchased from Sigma-Aldrich (Gillingham, UK). All chemicals were used as received without further purification. Distilled water was used to prepare all aqueous solutions.

### 2.2. Preparation of Periodate-Oxidized Lignin

Periodate-oxidized lignin, hereafter operationally designated as dialdehyde lignin (DAL), was prepared by periodate oxidation of alkali lignin. Briefly, sodium periodate (NaIO_4_; 15 g) was dissolved in 100 mL of a 15% water/ethanol solution, after which alkali lignin (10 g) was added to the reaction mixture. The NaIO_4_-to-lignin mass ratio was therefore 1.5:1. Because technical alkali lignin is a heterogeneous macromolecular material without a single defined molar mass or quantified content of periodate-reactive structural units, a chemically meaningful oxidant-to-lignin molar ratio could not be calculated from the available supplier specifications. The oxidation was carried out at room temperature under continuous magnetic stirring at 300 rpm for 24 h. The reaction vessel was protected from light with aluminum foil throughout the oxidation process to minimize photodecomposition of NaIO_4_.

Following oxidation, the reaction mixture was subjected to vacuum filtration. The collected solid was transferred to a beaker, dispersed in 20 mL of distilled water, thoroughly mixed, and recovered by vacuum filtration. This water-washing procedure was repeated three times using a fresh 20 mL portion of distilled water in each cycle. Subsequently, the solid was washed three times with 20 mL portions of 50% (*v*/*v*) aqueous ethanol using the same dispersion and vacuum-filtration procedure. The purified oxidized lignin was dried in an oven at 45 °C until a constant mass was obtained and was subsequently used as DAL for preparation of the bioplastic films.

The aldehyde-group content of the starting lignin and oxidized product was not quantitatively determined, and the isolated oxidation yield was not recorded. Accordingly, DAL is used here as an operational designation for the periodate-oxidized lignin, and the nominal Ch:DAL formulation ratios reported below represent mass ratios rather than stoichiometric NH_2_:CHO ratios. FTIR spectroscopy (Thermo Fisher Scientific, Madison, WI, USA) was used to assess changes consistent with oxidation and subsequent imine formation, but it did not provide quantitative determination of aldehyde functionality.

### 2.3. Preparation of Chitosan–Dialdehyde Lignin Bioplastic Films

Chitosan–dialdehyde lignin (Ch-DAL) bioplastic films were prepared through Schiff base interactions between the primary amino groups of chitosan and the aldehyde functionalities of DAL. Three formulations with nominal Ch:DAL mass ratios of 1:1, 2:1, and 1:2 were prepared. Their complete compositions are presented in Table 1.

The glycerol loading was calculated relative to the combined mass of chitosan and DAL using a glycerol density of 1.25 g mL^−1^. It was maintained at 17.9 wt% relative to the total Ch-DAL mass in all formulations. The nominal total formulation content was calculated from the combined amounts of chitosan, DAL, tartaric acid, and glycerol relative to the volume of tartaric acid solution.

Chitosan was first dissolved in a 3% (w/v) aqueous tartaric acid solution at 25 °C under continuous stirring at 600 rpm. DAL was subsequently incorporated into the chitosan solution, and the mixture was stirred at 40 °C and 500 rpm until a homogeneous viscous dispersion was obtained. Although different batch sizes were used, the volumes of tartaric acid solution and glycerol were proportionally adjusted. The total biopolymer concentration, calculated from the combined mass of chitosan and DAL relative to the volume of tartaric acid solution, was maintained at 4.0% (w/v) in all formulations. The nominal total formulation content, including chitosan, DAL, tartaric acid, and glycerol, was approximately 7.71% (w/v).

The resulting mixtures were cast into 9 × 9 cm molds and distributed uniformly across the mold surface. The cast films were dried in an oven at 45 °C for approximately 24 h, until they were completely dry and could be readily removed from the casting surface. After drying, the Ch-DAL films were carefully removed from the molds. Prior to characterization, the dried films were stored under ambient laboratory conditions at approximately 25 ± 2 °C for at least 24 h. Relative humidity was not specifically controlled.

### 2.4. Qualitative Assessment of Film Behavior in Different Media

The behavior of the Ch-DAL bioplastic films in different liquid media was qualitatively evaluated using the Ch:DAL 1:1 formulation. Film specimens measuring 1.0 × 0.6 cm and having a thickness of approximately 0.77 mm were individually immersed in 10 mL of distilled water (H_2_O), chloroform, absolute ethanol, 1 M sodium hydroxide (NaOH), 1 M hydrochloric acid (HCl), or dimethyl sulfoxide (DMSO) in sealed glass vials. The specimens were maintained at room temperature (approximately 25 °C) for 24 h. Following immersion, the films were visually examined for swelling, fragmentation, disintegration, dissolution, and loss of physical integrity. Changes in the appearance or coloration of the surrounding media were also recorded as qualitative indicators of material release. Because mass variation, swelling ratio, and soluble fraction were not measured, this experiment was considered a qualitative visual assessment rather than a quantitative solvent-resistance measurement.

### 2.5. Thermally Assisted Repair Test

The thermally assisted repair behavior of the Ch-DAL bioplastic films was evaluated using only the Ch:DAL 1:1 formulation and three independently prepared film specimens (n = 3). The 1:1 formulation was selected because it exhibited the highest initial tensile strength and Young’s modulus among the three formulations. A pressing temperature of 50 °C was selected to increase polymer-chain mobility and facilitate interfacial contact and dynamic imine-bond exchange while limiting the risk of thermal degradation or excessive structural alteration of the biopolymer film.

The original cast films measured approximately 9 × 9 cm. Each film specimen was completely cut into two separate pieces using a sharp blade, and the freshly cut surfaces were carefully realigned in direct contact. The cut was made perpendicular to the direction of the subsequently applied tensile force so that the repaired interface was directly subjected to loading during tensile testing. The assembled specimens were placed between Teflon sheets and hot-pressed at 50 °C for 10 min using a BOYN hot press. The applied pressure was not instrumentally controlled or quantitatively recorded during the original experiment. After treatment, the specimens were allowed to cool naturally to room temperature between the Teflon sheets and were then carefully removed from the press.

Rectangular tensile-test specimens were cut from the repaired film so that the repaired interface was positioned across the width of the specimen and approximately at the center of the gauge region. Three independently prepared repaired specimens were tested (n = 3). The repaired films were visually examined to assess restoration of structural continuity at the cut interface and were subsequently subjected to tensile testing. Their tensile strength, elongation at break, and Young’s modulus were compared with those of the corresponding uncut, pristine films that had not undergone hot pressing.

An additional uncut film subjected to the same hot-pressing conditions was not included in the original experimental design. Consequently, the observed changes in mechanical properties cannot be attributed exclusively to repair of the cut interface and may also reflect thermo-mechanically induced changes in the bulk film structure. Accordingly, the experiment is described as thermally assisted repair rather than quantitative self-healing. The specimen geometry, position of the repaired interface, and direction of tensile loading are illustrated schematically in Figure S1.

### 2.6. Thermo-Mechanical Reprocessing

The thermo-mechanical reprocessability of the Ch-DAL bioplastic films was evaluated for the Ch:DAL 1:1 and 2:1 formulations through two successive fragmentation and hot-pressing cycles. Pristine films were cut into small pieces and hot-pressed between Teflon sheets at 50 °C for 10 min using a BOYN hot press (Model BYP-15, Hangzhou BOYN Instrument Co., Ltd., Hangzhou, China). The applied pressure, initial amount of processed material, and final dimensions of the regenerated films were not quantitatively recorded during the original experiment and therefore cannot be reported retrospectively. Following hot pressing, the regenerated films were allowed to cool naturally to room temperature between the Teflon sheets and were then carefully recovered.

After each reprocessing cycle, the regenerated films were fragmented again and subjected to the same thermo-mechanical treatment. The materials were designated as cycle 0 (pristine), cycle 1 (second-generation film), and cycle 2 (third-generation film). Following each cycle, rectangular test specimens were cut from the regenerated films and analyzed using the same uniaxial tensile-testing procedure applied to the pristine films. Three independently prepared specimens were tested for each formulation and reprocessing stage (n = 3). Tensile strength, elongation at break, and Young’s modulus were determined and compared with those of the pristine material to evaluate changes in mechanical performance during repeated reprocessing.

The Ch:DAL 1:2 formulation was excluded from repeated reprocessing because its low initial tensile strength and Young’s modulus produced a mechanically weak film that could not be handled and tested reliably after repeated fragmentation and hot pressing. In addition, the mass recovered after each cycle was not recorded. Therefore, the formation of a continuous film after hot pressing is interpreted as evidence of reprocessability, but not as evidence of complete quantitative material recovery or closed-loop recycling.

### 2.7. Characterization

#### 2.7.1. Fourier Transform Infrared (FTIR) Spectroscopy

Fourier transform infrared (FTIR) spectroscopy was used to characterize the chemical functionalities and interactions within the Ch–DAL bioplastic films. The dried film specimens were analyzed directly without dissolution, dilution, chemical pretreatment, or preparation as KBr pellets. FTIR spectra were recorded using a Cary 660 FTIR spectrometer (Agilent Technologies, Santa Clara, CA, USA) over the spectral range of 4000–700 cm^−1^ at a resolution of 4 cm^−1^. Each spectrum was obtained from 16 scans in absorbance mode. The specific sampling accessory and acquisition geometry used during the original measurements were not documented and therefore cannot be stated retrospectively. The resulting spectra were plotted and analyzed using Origin 2024b software (version 10.1.5.132; OriginLab Corporation, Northampton, MA, USA).

#### 2.7.2. Thermogravimetric Analysis

Thermogravimetric analysis (TGA) was performed using a LABSYS evo thermogravimetric analyzer (SETARAM, Caluire-et-Cuire, France) to evaluate the thermal behavior of the bioplastic films. Samples weighing 8–10 mg were heated from 30 to 600 °C at a heating rate of 10 °C min^−1^ under a nitrogen atmosphere. The resulting thermogravimetric curves were plotted and analyzed using Origin 2024b software (version 10.1.5.132; OriginLab Corporation, Northampton, MA, USA).

#### 2.7.3. Tensile Testing and Mechanical Characterization

The mechanical properties of the Ch–DAL bioplastic films were evaluated using a TA.XT Plus texture analyzer (Stable Micro Systems, Godalming, UK) equipped with a tensile testing fixture and a 30 kg load cell. Because the available films were smaller than the standard specimen geometries specified in ASTM D638-22 [24] and ISO 527-1:2019 [25], an adapted in-house tensile-testing procedure was used. Therefore, the measurements were not considered fully compliant with either standard.

Rectangular specimens measuring approximately 50 mm in length and 10 mm in width were cut from the films using a sharp blade. Film thickness was measured at five locations using a digital micrometer, and the mean thickness of approximately 0.77 mm was used to calculate the initial cross-sectional area. The specimens were mounted between the grips with an initial gauge length of 30 mm and subjected to uniaxial tensile loading at room temperature using a crosshead speed of 2 mm s^−1^. Three independently prepared specimens were tested for each formulation and processing condition (n = 3).

Engineering stress was calculated by dividing the measured force by the initial cross-sectional area of each specimen. Engineering strain was calculated as the change in grip separation divided by the initial gauge length. Tensile strength was defined as the maximum engineering stress, while elongation at break was determined from the engineering strain at specimen failure. For graphical presentation, engineering strain was expressed as a percentage. For calculation of Young’s modulus, percentage strain values were converted to dimensionless values by dividing them by 100. Young’s modulus was then calculated separately for each specimen by linear regression of engineering stress against dimensionless engineering strain over the initial linear region between 0% and 2% strain. The resulting regression slope was expressed in MPa. Young’s modulus values are reported as the mean ± standard deviation of three independently prepared specimens for each formulation (n = 3). The same tensile-testing procedure was applied to pristine, thermally repaired, and thermo-mechanically reprocessed films to enable comparison of their mechanical performance.

### 2.8. Statistical Analysis

Mechanical-property data are presented as the mean ± standard deviation (SD) of three independently prepared and tested specimens for each formulation or processing condition (n = 3). Statistical comparisons between the pristine and thermally repaired Ch-DAL 1:1 films were performed separately for tensile strength, elongation at break, and Young’s modulus using a two-tailed unpaired Student’s *t*-test. Differences were considered statistically significant at *p* < 0.05. Statistical calculations and graphical presentation were performed using Origin 2024b software (version 10.1.5.132; OriginLab Corporation, Northampton, MA, USA). Because the thermo-mechanical reprocessing experiments were exploratory and included only three specimens per processing cycle, changes across successive reprocessing cycles were evaluated descriptively and no inferential statistical significance was claimed. FTIR, TGA/DTG, and film behavior in different liquid media were also evaluated descriptively or qualitatively, as applicable.

## 3. Results

To investigate the formation of the Ch-DAL bioplastic network, the sequential processes of lignin oxidation, chitosan protonation, and subsequent cross-linking between chitosan and dialdehyde lignin (DAL) through Schiff base interactions were examined (Figure 1). Alkali lignin was first oxidized with sodium periodate to introduce aldehyde functionalities and obtain DAL (Figure 1a). Chitosan was subsequently protonated with tartaric acid to facilitate its dissolution and provide reactive amino groups for subsequent network formation (Figure 1b). The aldehyde groups of DAL then interacted with the primary amino groups of chitosan, enabling the formation of imine (C=N) linkages through Schiff base chemistry and the development of the Ch-DAL network (Figure 1c). The experimental procedure, including chitosan dissolution in tartaric acid solution, incorporation of DAL, and formation of the final Ch-DAL bioplastic film, is summarized schematically (Figure 2).

To evaluate the chemical interactions involved in Ch-DAL network formation, the FTIR spectra of chitosan (Ch), dialdehyde lignin (DAL), glycerol, and the Ch-DAL bioplastic were compared (Figure 3). The Ch-DAL bioplastic showed a broad O–H/N–H stretching band centered at 3374 cm^−1^, compared with bands at 3340 cm^−1^ for chitosan and 3320 cm^−1^ for glycerol. The shift and broadening of this band indicate changes in hydrogen-bonding interactions among the components of the bioplastic. A carbonyl absorption was observed at 1732 cm^−1^ in the Ch-DAL spectrum, close to the aldehyde-associated band of DAL at 1729 cm^−1^. The aromatic skeletal vibration of DAL at 1577 cm^−1^ remained detectable after incorporation into the Ch-DAL film. The bioplastic also retained the characteristic polysaccharide region, including the band at 1068 cm^−1^ attributed mainly to C–O and C–O–C vibrations. A band observed near 1634 cm^−1^ may include overlapping contributions from C=N stretching associated with imine linkages and vibrations in the chitosan amide I region. Therefore, this band was considered consistent with and supportive of Schiff base formation between the aldehyde groups of DAL and the available primary amino groups of chitosan, rather than definitive evidence of imine formation. The band near 1541 cm^−1^ was attributed primarily to N–H bending and C–N stretching contributions within the chitosan amide II region. Overall, the FTIR results support the proposed formation of imine linkages and changes in hydrogen-bonding interactions within the Ch-DAL network; however, FTIR alone does not directly identify or quantify the extent of imine cross-linking.

To determine how the Ch:DAL composition influences the mechanical behavior of the bioplastics, tensile strength, elongation at break, and Young’s modulus were compared among the three formulations (Figure 4 and Figure 5 and Table 2). The BP 1:1 formulation exhibited the highest tensile strength (8.85 MPa) and Young’s modulus (118.7 MPa), with an elongation at break of 19%, indicating a comparatively strong and rigid material. This mechanical response may hypothetically be associated with a more favorable balance between the amino groups of chitosan and the aldehyde groups of DAL. However, because the aldehyde content and the NH_2_ molar ratio were not determined, the proposed relationship cannot be confirmed from the present results. Increasing the chitosan content (BP 2:1) markedly altered the mechanical response: the tensile strength decreased to 1.27 MPa, whereas the elongation at break increased to 238%. The greater extensibility of this formulation may reflect increased mobility of chitosan chains that are less constrained by imine-mediated network interactions. Increasing the DAL content produced a similarly pronounced but distinct change in mechanical behavior. BP 1:2 exhibited the lowest tensile strength (0.27 MPa) and Young’s modulus (0.288 MPa), while maintaining a high elongation at break (178%). One possible explanation is that excess DAL may produce compositional heterogeneity or lignin- or aldehyde-rich regions that reduce effective network connectivity; however, these structural features were not directly evaluated and therefore remain hypothetical. Thus, the mechanical data identify the 1:1 Ch:DAL ratio as the composition with the highest strength and stiffness, whereas either chitosan- or DAL-rich formulations favor substantially softer and more extensible behavior. Together with the FTIR results, these composition-dependent changes are consistent with a possible contribution of imine-mediated interactions to the mechanical response of the resulting network. Nevertheless, the present data neither establish an optimal NH_2_ stoichiometric balance nor directly quantify imine content or cross-link density.

Values are presented as mean ± standard deviation (SD) from three independently prepared specimens for each formulation (n = 3).

To evaluate the solvent resistance and chemical stability of the Ch-DAL bioplastics, the films were exposed to water, chloroform, ethanol, DMSO, 1 M NaOH, and 1 M HCl (Figure 6). The films retained their structural integrity in water, chloroform, ethanol, and DMSO, with no visible dissolution and only limited swelling. Similar behavior was observed in 1 M NaOH, where the films remained intact during the test period. A slight yellow coloration developed in the alkaline solution, suggesting the release of a small amount of soluble or weakly associated lignin-derived material from the polymer matrix. The preservation of film integrity across these media is consistent with the presence of the interconnected Ch-DAL network supported by imine linkages and intermolecular interactions.

A distinctly different response was observed in 1 M HCl, where the films underwent rapid fragmentation and loss of structural integrity (Figure 6). Under acidic conditions, protonation of the amino groups of chitosan can increase electrostatic repulsion and promote swelling, while acid-mediated hydrolysis of the imine (C=N) linkages can disrupt the Ch-DAL network. The pronounced instability in HCl, compared with the structural stability observed in the other tested media, indicates a strong dependence of the Ch-DAL network on the chemical environment. This acid-responsive behavior may be relevant to applications in which controlled disintegration of the material is desirable.

Thermogravimetric analysis revealed distinct multistage degradation profiles for alkali lignin, DAL, and the 1:1 Ch-DAL bioplastic film (Figure 7a,b). All samples exhibited initial mass loss below approximately 150 °C. Alkali lignin showed a broad degradation profile between approximately 150 and 500 °C, with overlapping DTG events at approximately 130, 280, and 490 °C and a residual mass of approximately 17% at 600 °C. DAL exhibited its principal degradation region between approximately 200 and 310 °C, with the main DTG minimum at approximately 285–290 °C and a residual mass of approximately 57% at 600 °C. The 1:1 Ch–DAL film showed a major mass-loss stage between approximately 200 and 310 °C, with a principal DTG minimum at approximately 250 °C, an additional shoulder at approximately 280–290 °C, and a residual mass of approximately 33% at 600 °C. These results demonstrate distinct thermal-degradation profiles and residue formation among alkali lignin, DAL, and the multicomponent Ch-DAL film.

To evaluate the thermally assisted repair behavior of the Ch-DAL bioplastic, the film was completely cut into two pieces, the fractured surfaces were brought into contact, and the sample was subjected to thermal pressing at 50 °C. After treatment, the separated pieces rejoined to form a continuous film that could be handled without visible separation at the repaired interface (Figure 8). This macroscopic recovery indicates that the Ch-DAL network retained sufficient mobility under the applied thermal and mechanical conditions to re-establish physical continuity across the damaged region. However, this visual repair does not demonstrate recovery of the original mechanical properties.

To determine how the healing treatment affected mechanical performance, the tensile properties of the original and healed Ch-DAL films were compared (Figure 9 and Table 3). The original film exhibited a tensile strength of 8.85 ± 0.09 MPa, an elongation at break of 19.0 ± 0.1%, and a Young’s modulus of 118.7 ± 0.7 MPa. After thermal repair, the tensile strength increased to 12.9 ± 0.11 MPa, Young’s modulus increased to 316.9 ± 1.6 MPa, and elongation at break decreased to 4.3 ± 0.05%. A two-tailed unpaired Student’s *t*-test indicated statistically significant differences between the original and thermally repaired films in tensile strength (*p* < 0.0001), Young’s modulus (*p* < 0.0001), and elongation at break (*p* < 0.0001). These results indicate that thermal pressing was associated with significantly increased strength and stiffness but significantly reduced extensibility.

The recovery of film continuity is consistent with rearrangement of the dynamic Ch-DAL network during thermal pressing. Reversible exchange of imine linkages at the damaged interface may facilitate reconnection of the polymer network, while residual aldehyde and amino groups may undergo additional reactions during treatment. The higher tensile strength and Young’s modulus after healing may also reflect increased intermolecular association or network densification during hot pressing. These structural changes would account for the increased resistance to deformation and the corresponding reduction in elongation at break observed after treatment.

To examine the thermo-mechanical reprocessability of the Ch-DAL bioplastic, the 1:1 formulation was subjected to consecutive reprocessing cycles involving mechanical fragmentation and hot pressing. The pristine film was first cut into small fragments and consolidated by thermal pressing to produce a second-generation (2nd) film; the recovered film was then fragmented and processed again under the same conditions to obtain a third-generation (3rd) film (Figure 10). After each reprocessing cycle, the fragmented material could be consolidated into a continuous film, demonstrating its remolding capability. However, the ability to reform a continuous film should not be interpreted as recovery of the original mechanical properties. Changes in film appearance and compaction were observed across successive cycles, suggesting that repeated fragmentation and thermal pressing altered the organization of the material. To determine how these processing-induced changes affected mechanical performance, the pristine, 2nd-, and 3rd-generation films were subsequently compared by tensile testing, with tensile strength, elongation at break, and Young’s modulus used to characterize the evolution of mechanical behavior during reprocessing.

To evaluate changes in mechanical performance during successive reprocessing cycles, the tensile properties of the pristine, second-generation (2nd), and third-generation (3rd) 1:1 Ch–DAL films were compared (Figure 11 and Table 4). The pristine film exhibited a tensile strength of 8.85 MPa, an elongation at break of 19%, and a Young’s modulus of 118.7 MPa. After the first reprocessing cycle, the tensile strength decreased to 1.87 MPa, representing a reduction of approximately 79% relative to the pristine film, accompanied by a decrease in elongation at break to 13% and a Young’s modulus of 28.7 MPa. The third-generation film showed partial recovery of tensile strength to 3.10 MPa and a Young’s modulus of 75.85 MPa, while elongation at break decreased further to 11%. Despite this partial improvement relative to the second-generation film, the tensile strength of the third-generation film remained approximately 65% below that of the pristine film. Thus, the material retained its capacity for repeated remolding but did not recover its original mechanical performance. These findings distinguish physical reprocessability from mechanical-property retention.

The initial decrease in tensile strength and Young’s modulus may reflect changes in network organization during fragmentation and hot pressing, including incomplete fusion between film fragments and possible redistribution or loss of glycerol and residual moisture. The partial recovery observed in the third-generation film suggests further rearrangement of the Ch-DAL network during repeated thermal treatment. Additional interactions between residual functional groups, closer packing of polymer chains, or stronger intermolecular association could contribute to the increase in strength and stiffness relative to the second-generation film. The progressive decrease in elongation at break from 19% to 13% and 11%, however, indicates reduced deformability with successive processing. These changes show that repeated thermo-mechanical treatment modifies the mechanical response of the Ch-DAL network while preserving its capacity to be reprocessed into continuous films.

Values are presented as mean ± standard deviation (SD) from three independently prepared and tested specimens per reprocessing cycle (n = 3).

To evaluate the effect of repeated thermo-mechanical processing on the chitosan-rich formulation, the mechanical properties of pristine, second-generation (2nd), and third-generation (3rd) 2:1 Ch-DAL films were compared (Figure 12 and Table 5). The pristine film exhibited a tensile strength of 1.27 MPa, an elongation at break of 238%, and a Young’s modulus of 0.570 ± 0.002 MPa, reflecting its highly extensible mechanical behavior. After the first reprocessing cycle, the tensile strength decreased to 0.98 MPa, and the elongation at break decreased to 128%, while the Young’s modulus increased to 1.223 ± 0.002 MPa. Further reprocessing produced a third-generation film with a tensile strength of 0.84 MPa and an elongation at break of 100%, accompanied by a further increase in Young’s modulus to 1.470 ± 0.002 MPa. Thus, successive reprocessing produced a progressive reduction in tensile strength and extensibility while increasing the stiffness of the 2:1 formulation.

The pronounced decrease in elongation at break from 238% in the pristine film to 100% after two reprocessing cycles indicates a substantial reduction in chain mobility during repeated hot pressing. This change, together with the approximately 2.6-fold increase in Young’s modulus, is consistent with progressive reorganization of the polymer network. Repeated thermal treatment may promote closer intermolecular association or additional interactions among residual reactive groups, while redistribution or partial loss of glycerol and bound water may further restrict polymer-chain mobility. Despite these changes in mechanical response, the fragmented 2:1 material remained sufficiently processable to be consolidated into continuous films through successive thermo-mechanical cycles.

Values are presented as mean ± standard deviation (SD) from three independently prepared and tested specimens per reprocessing cycle (n = 3).

## 4. Discussion

Recent advances in bio-based covalent adaptable networks have shown that dynamic covalent chemistry can overcome the conventional trade-off between network stability and end-of-life processability. Chitosan-based polyimine vitrimers formed through Schiff base reactions have demonstrated high mechanical performance together with self-healing, solvent resistance, reprocessability, and acid-triggered degradation [24], while fully bio-based chitosan-polysaccharide polyimine networks have extended this concept to materials that are simultaneously self-healable, weldable, malleable, and recyclable [25]. Related lignin-derived systems further demonstrate the broader potential of dynamic networks for circular materials: lignin-containing covalent adaptable networks can achieve high mechanical strength while retaining substantial mechanical performance after thermal reshaping [26], and lignocellulosic vitrimer systems have been developed with repeated reprocessing and closed-loop reprocessing capabilities [27]. Dynamic aldehyde-derived networks based on alternative exchange chemistries likewise illustrate how reversible covalent interactions can couple mechanical integrity with adaptability and chemically triggered degradation [28]. Self-healing behavior has also been achieved in chitosan networks cross-linked with dialdehyde polysaccharides, supporting the broader applicability of reversible imine chemistry to renewable polymer systems [29]. Similarly, fully bio-based Schiff base vitrimers have demonstrated that imine-rich networks can undergo repeated reprocessing while maintaining a substantial fraction of their original mechanical performance [30]. Against this background, the present Ch-DAL system extends dynamic covalent design directly to periodate-oxidized lignin as a multifunctional macromolecular cross-linking component. Rather than examining mechanical performance, healing, or reprocessability as isolated functions, the present study shows that variation in the Ch:DAL ratio produces markedly different mechanical states and that the resulting films retain acid-responsive behavior, thermal repairability, and the capacity for successive thermo-mechanical reprocessing. These findings suggest that network composition controls not only the initial strength-ductility balance but also the adaptive behavior of the material after damage and repeated processing, providing preliminary guidance for the design of Ch-DAL films with different mechanical and processing characteristics Ch-DAL networks according to both mechanical and circularity requirements.

The FTIR results provide spectroscopic support for the formation of an imine-containing Ch-DAL network and are consistent with the characteristic changes reported for chitosan cross-linked with biobased aldehyde compounds. In the present system, the appearance of a band at 1634 cm^−1^, assigned to C=N stretching, together with the carbonyl absorption at 1732 cm^−1^ and changes in the O–H/N–H stretching region, indicates reaction between the primary amino groups of chitosan and aldehyde functionalities introduced into lignin by periodate oxidation. Comparable spectral changes have been reported for dialdehyde cellulose–chitosan hydrogels, where the aldehyde C=O band at 1716 cm^−1^ disappeared after reaction and a new imine band appeared at 1631 cm^−1^ [31]. The close correspondence between the C=N bands at 1631 cm^−1^ in the dialdehyde cellulose system and 1634 cm^−1^ in the present Ch-DAL films supports, but does not conclusively establish, the contribution of Schiff-base linkages to this spectral region. Similar evidence has been obtained in chitosan films modified with natural phenolic aldehydes, where FTIR and XPS confirmed conjugation through dynamic imine bonds [32], and in syringaldehyde-modified chitosan, in which Schiff base formation was likewise established spectroscopically [33]. More complex polysaccharide systems further show that the extent and consequences of imine formation depend strongly on the availability and balance of aldehyde and amino functionalities. Chitosan cross-linked with oxidized guar gum exhibited composition-dependent cross-linking as the NH_2_/CHO ratio was varied, with FTIR and XPS evidence interpreted together with rheological, swelling, degradation, and mechanical measurements [34], while ozone-generated carbonyl groups in starch-chitosan systems produced Schiff base cross-links whose formation was supported by both FTIR and NMR analysis [35]. In the Ch-DAL films, the shift and broadening of the O–H/N–H band to 3374 cm^−1^ additionally suggest reorganization of hydrogen-bonding interactions among chitosan, DAL, glycerol, and tartaric acid, consistent with the O–H shift reported following formation of dialdehyde cellulose-chitosan networks [31]. Retention of the lignin aromatic vibration at 1577 cm^−1^ further indicates incorporation of the aromatic DAL framework rather than complete disruption of its underlying structure. These spectral features therefore support a network in which covalent imine linkages coexist with extensive noncovalent interactions; however, the presence and intensity of FTIR bands alone cannot establish the absolute cross-link density of the material. Consequently, the composition-dependent mechanical behavior observed in the Ch-DAL films should be interpreted as indirect structure-property evidence for differences in network organization rather than as a direct measurement of imine cross-link density.

The pronounced dependence of mechanical behavior on the Ch:DAL ratio indicates that composition plays a central role in controlling the balance between network connectivity and polymer-chain mobility. Among the three formulations, the 1:1 Ch:DAL film exhibited the highest tensile strength (8.85 MPa) and Young’s modulus (118.7 MPa), with a comparatively low elongation at break (19%), whereas increasing the chitosan content to 2:1 reduced the tensile strength to 1.27 MPa while increasing elongation to 238%; the DAL-rich 1:2 formulation was similarly soft and extensible, with a tensile strength of 0.27 MPa and elongation of 178%. Such non-linear composition-property relationships are characteristic of dynamic imine-crosslinked biopolymer networks, in which increasing the concentration of reactive groups does not necessarily produce a mechanically stronger material. Zhou et al. similarly found no linear relationship between aldehyde content and tensile strength in dialdehyde starch-diamine networks, with an intermediate aldehyde content of 41.1% producing the highest tensile strength of 27.8 MPa [36]. The mechanical response also depends on the contribution of reversible noncovalent interactions: in soy protein-dialdehyde starch films, Schiff base cross-linking increased network strength, whereas lignosulfonate-mediated sacrificial hydrogen bonding substantially increased extensibility, with strain at break reaching 153% [37]. Comparable effects of aldehyde availability have been observed in chitosan-based systems, where excessive aldehyde modification produced localized aggregation, reduced network interconnectivity, and lower mechanical stiffness rather than continued reinforcement [38,39]. However, such compositional heterogeneity was not directly evaluated in the present study. Conversely, the exceptionally high elongation of the 2:1 formulation is consistent with a larger population of chitosan chains remaining less constrained by imine cross-links, allowing greater segmental mobility before failure. The importance of balancing cross-linking and molecular mobility is further demonstrated by dialdehyde starch films, where changing diamine structure and molar ratio shifted the mechanical response between high strength (62.9 MPa) and high extensibility (126.2%) [40]. Chitosan–lignin films likewise show that lignin content must be optimized rather than simply maximized, with an optimized composition producing 2.82- and 6.26-fold increases in tensile strength and Young’s modulus, respectively [41]. Similarly, vanillin-derived dialdehyde cross-linking has been reported to increase the fracture stress of chitosan films by 3.8–4.5-fold [38]. Taken together, these comparisons suggest that the superior strength and stiffness observed for the 1:1 Ch:DAL film may be associated with differences in imine-mediated connectivity, hydrogen bonding, and chain mobility. However, these mechanisms were not directly quantified. Nevertheless, because the Ch:DAL ratios used here are mass feeding ratios rather than measured NH_2_:CHO stoichiometric ratios, the superior performance of the 1:1 formulation should not be interpreted as evidence of stoichiometrically optimal cross-linking without direct determination of aldehyde content and cross-link density.

The solvent-resistance profile of the Ch-DAL films further reflects the combined contribution of dynamic imine cross-links and noncovalent interactions to network stability. The films retained their structural integrity in water, ethanol, chloroform, DMSO, and 1 M NaOH, whereas exposure to 1 M HCl resulted in rapid fragmentation and loss of film integrity. Similar stability has been observed in dynamically cross-linked cellulose networks, where imine formation between amino-functionalized cellulose and divanillin converted the initially water-soluble components into networks with pronounced resistance to water and organic solvents [41,42,43,44]. The stability of the present Ch-DAL films under neutral and alkaline conditions is therefore consistent with formation of an interconnected network that limits polymer-chain solubilization, with hydrogen bonding and lignin-associated interactions likely providing additional stabilization. Lignin-containing chitosan hydrogels likewise exhibit extensive intermolecular and noncovalent interactions in addition to covalent network formation, contributing to their structural stability while retaining stimulus-responsive behavior [42]. In contrast, the rapid disruption of the Ch-DAL films in strong acid is consistent with the reversible character of imine chemistry. Dynamic imine-crosslinked dextran-chitosan networks have been shown to undergo acid-induced network dissociation, with protonation of chitosan contributing to their low-pH response [44], while aldehyde-functionalized cellulose/chitosan networks exhibit accelerated imine hydrolysis and increased molecular release under acidic conditions [45]. Particularly relevant is the divanillin-crosslinked cellulose system, in which imine hydrolysis under acidic conditions enabled recovery of the original amino- and aldehyde-containing building blocks for subsequent network reconstruction [43]. In the present Ch-DAL system, protonation of chitosan amino groups under strongly acidic conditions may increase electrostatic repulsion and hydration of the polymer chains, while acid-catalyzed hydrolysis of C=N linkages may progressively reduce network connectivity. The observed acid sensitivity should therefore not be regarded simply as poor chemical resistance, but as an intrinsic consequence of the reversible chemistry responsible for the adaptive character of the material. This combination of stability under neutral, alkaline, and selected organic-solvent conditions with pronounced acid-triggered disassembly may be advantageous for applications in which structural integrity during use is required together with chemically accessible degradation or end-of-life processing.

The TGA and DTG results further demonstrated distinct thermal responses among alkali lignin, DAL, and the 1:1 Ch-DAL bioplastic film. Alkali lignin produced the lowest residual mass at 600 °C (approximately 17%), whereas DAL retained approximately 57%. The higher residue of DAL may reflect increased char formation following periodate oxidation; however, a contribution from residual inorganic sodium-containing species cannot be excluded. The Ch-DAL film exhibited its principal DTG minimum at approximately 250 °C and retained approximately 33% of its initial mass at 600 °C, intermediate between alkali lignin and DAL. Because the film contained chitosan, DAL, glycerol, and tartaric acid, its thermal events represent overlapping degradation processes and cannot be attributed exclusively to imine cross-linking. Therefore, these results demonstrate a distinct thermal-degradation profile for the Ch-DAL formulation but do not, by themselves, establish improved thermal stability or quantify cross-link density.

The thermally assisted repair treatment re-established macroscopic continuity in the 1:1 Ch-DAL film but produced a mechanically distinct material. Following hot pressing at 50 °C, tensile strength increased from 8.85 ± 0.09 to 12.9 ± 0.11 MPa and Young’s modulus increased from 118.7 ± 0.7 to 316.9 ± 1.6 MPa, while elongation at break decreased markedly from 19.0 ± 0.1% to 4.3 ± 0.05%. Thermally assisted repair in imine-containing networks has been associated with molecular mobility, interfacial contact, reversible imine exchange, and hydrogen-bond reorganization [43,46,47]. Therefore, the macroscopic continuity observed in the present film is consistent with possible interfacial network reorganization. However, these mechanisms were not directly evaluated. The increased strength and stiffness, together with the pronounced reduction in elongation, suggest that hot pressing may also have altered the bulk network. Possible contributions from additional aldehyde–amine interactions, hydrogen-bond rearrangement, closer chain packing, and physical densification remain hypothetical. Consequently, the increase in post-repair strength should not be interpreted as a healing efficiency above 100% or as complete recovery of the original mechanical state. Successive thermo-mechanical reprocessing produced distinctly different mechanical trajectories in the 1:1 and 2:1 Ch-DAL formulations, suggesting that initial network composition may influence the response to reprocessing. In the 1:1 film, tensile strength decreased sharply from 8.85 to 1.87 MPa after the first cycle but partially recovered to 3.10 MPa after the second, whereas the 2:1 film showed a progressive decline from 1.27 to 0.98 and 0.84 MPa. Dynamic imine networks can undergo bond exchange and structural rearrangement during hot pressing, allowing damaged or fragmented materials to be reformed [1,14,17]. The partial recovery of the 1:1 formulation may reflect renewed interfacial bonding, additional cross-link formation, or improved chain packing during the second cycle. By contrast, the progressive loss of ductility in the 2:1 formulation, together with its increasing modulus, suggests gradual restriction of chain mobility, potentially associated with network rearrangement and redistribution or loss of glycerol and bound water. These proposed mechanisms remain hypothetical because cross-link formation, chain packing, and changes in glycerol or water content were not directly evaluated.

The visible cracks, voids, and uneven thickness of the reprocessed films suggest that the applied pressing conditions did not produce specimens with the same physical quality as the pristine films. These defects may have contributed to the reduced mechanical properties. Because the applied pressure was not quantitatively recorded, the effects of pressing temperature, pressure, and time could not be systematically evaluated. Future studies should optimize these parameters and apply FTIR and TGA/DTG analyses after each reprocessing cycle to evaluate possible chemical or thermal degradation.

The combination of renewable chitosan and lignin-derived components with repairability and thermo-mechanical reprocessability supports the potential of Ch-DAL films as adaptable biobased materials, but the present findings do not yet establish suitability for practical packaging or closed-loop circular use. Chitosan films cross-linked with biobased aldehydes have shown substantial improvements in mechanical strength, barrier performance, and food-preservation functionality [3,9], while lignin incorporation can enhance tensile properties, water-vapor resistance, and bioactive performance [12]. The current study did not quantify oxygen or water-vapor permeability, biodegradation, compostability, migration behavior, or long-term environmental stability, and solvent resistance was assessed qualitatively. In addition, FTIR supports imine formation but does not directly quantify cross-link density [2,5,6]. Future studies should therefore combine quantitative barrier and degradation testing with aldehyde-content analysis, cross-link-density measurements, additional reprocessing cycles, and systematic evaluation of mechanical retention before claims concerning packaging performance, biodegradability, or circularity are strengthened.

## 5. Conclusions

This study demonstrates the preparation of Ch-DAL bioplastic films with composition-dependent mechanical properties, thermally assisted repair behavior, and thermo-mechanical reprocessability. FTIR results are consistent with the formation of imine linkages and the presence of hydrogen-bonding interactions; however, aldehyde content, degree of conversion, and cross-link density were not quantitatively determined. Therefore, the available results support, but do not establish, the proposed dynamic Schiff-base network structure.

The balance between chitosan and dialdehyde lignin influenced the mechanical response of the films. However, because the investigated Ch ratios were mass ratios rather than measured NH_2_ stoichiometric ratios, the superior mechanical performance of the 1:1 formulation should not be interpreted as evidence of stoichiometrically optimal cross-linking.

The observed repair and reprocessing behavior is consistent with network reorganization that may be facilitated by dynamic imine linkages and hydrogen-bonding interactions. Thermal pressing restored macroscopic continuity across the damaged interface but produced a mechanically distinct repaired material rather than restoring the original mechanical state. Moreover, repeated damage–repair cycles and long-term durability were not evaluated; therefore, the present results do not establish self-healing efficiency or extended service life.

The films could be fragmented and remolded through successive thermo-mechanical reprocessing cycles. Nevertheless, remolding was accompanied by substantial changes and losses in mechanical performance, demonstrating that reprocessability is not equivalent to recovery of the original properties.

The use of renewable chitosan and lignin-derived components supports the potential of these films as adaptable bio-based polymer materials. However, their environmental sustainability cannot be established from the present results because life-cycle assessment, biodegradation, compostability, and environmental-impact analyses were not performed. Future studies should quantify aldehyde content, degree of imine conversion, and cross-link density and should evaluate repeated damage-repair behavior, long-term durability, mechanical-property retention, biodegradation, compostability, and overall environmental impact.

## Figures and Tables

**Figure 1:**
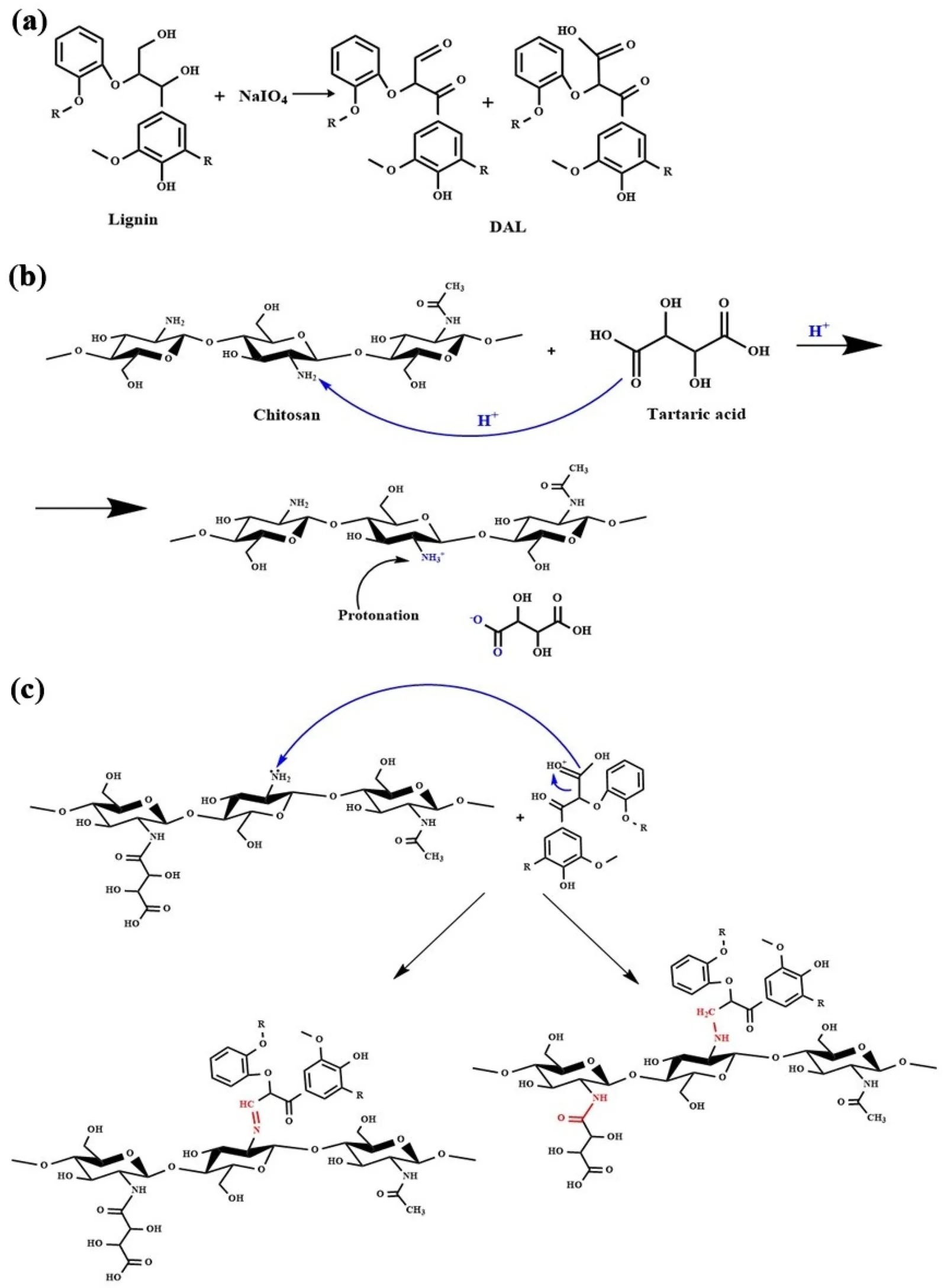
Proposed schematic representation of (**a**) sodium periodate oxidation of representative lignin structural motifs, (**b**) protonation of chitosan by tartaric acid, and (**c**) possible formation of imine (C=N) linkages between chitosan and dialdehyde lignin through Schiff base interactions. Black arrows indicate the proposed sequence of the reaction steps, while blue curved arrows indicate proton transfer and the proposed interactions between the reactive functional groups. Red bonds and atoms highlight the proposed newly formed imine linkages. The structures are simplified representations because of the inherent structural heterogeneity of lignin.

**Figure 2:**
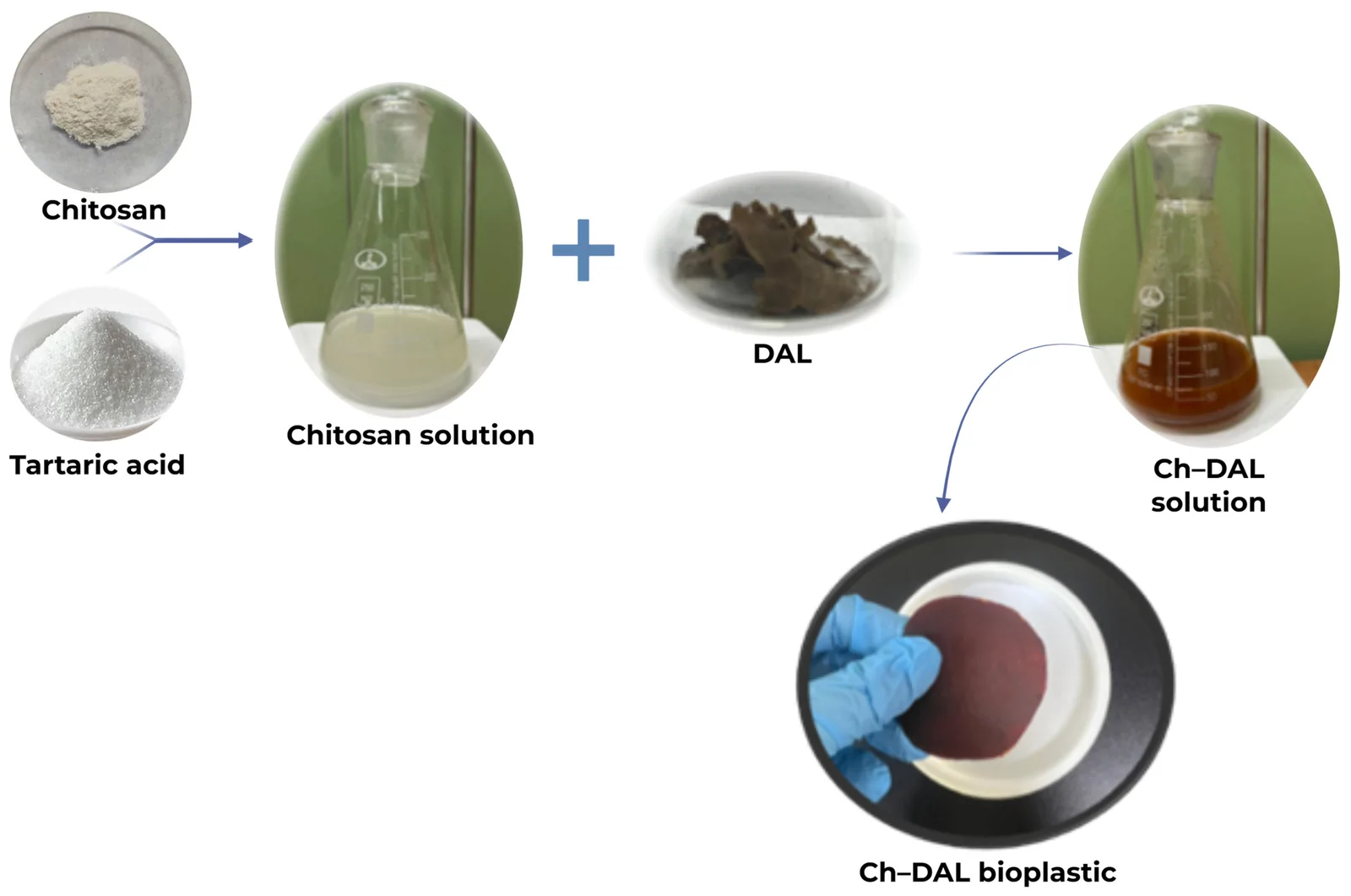
Schematic illustration of the experimental preparation of the Ch-DAL bioplastic, including dissolution and protonation of chitosan in tartaric acid solution, addition of dialdehyde lignin (DAL), formation of the Ch-DAL solution, and production of the final Ch-DAL bioplastic film. The photographs show the actual materials, intermediate solutions, and final film prepared and used in this study.

**Figure 3:**
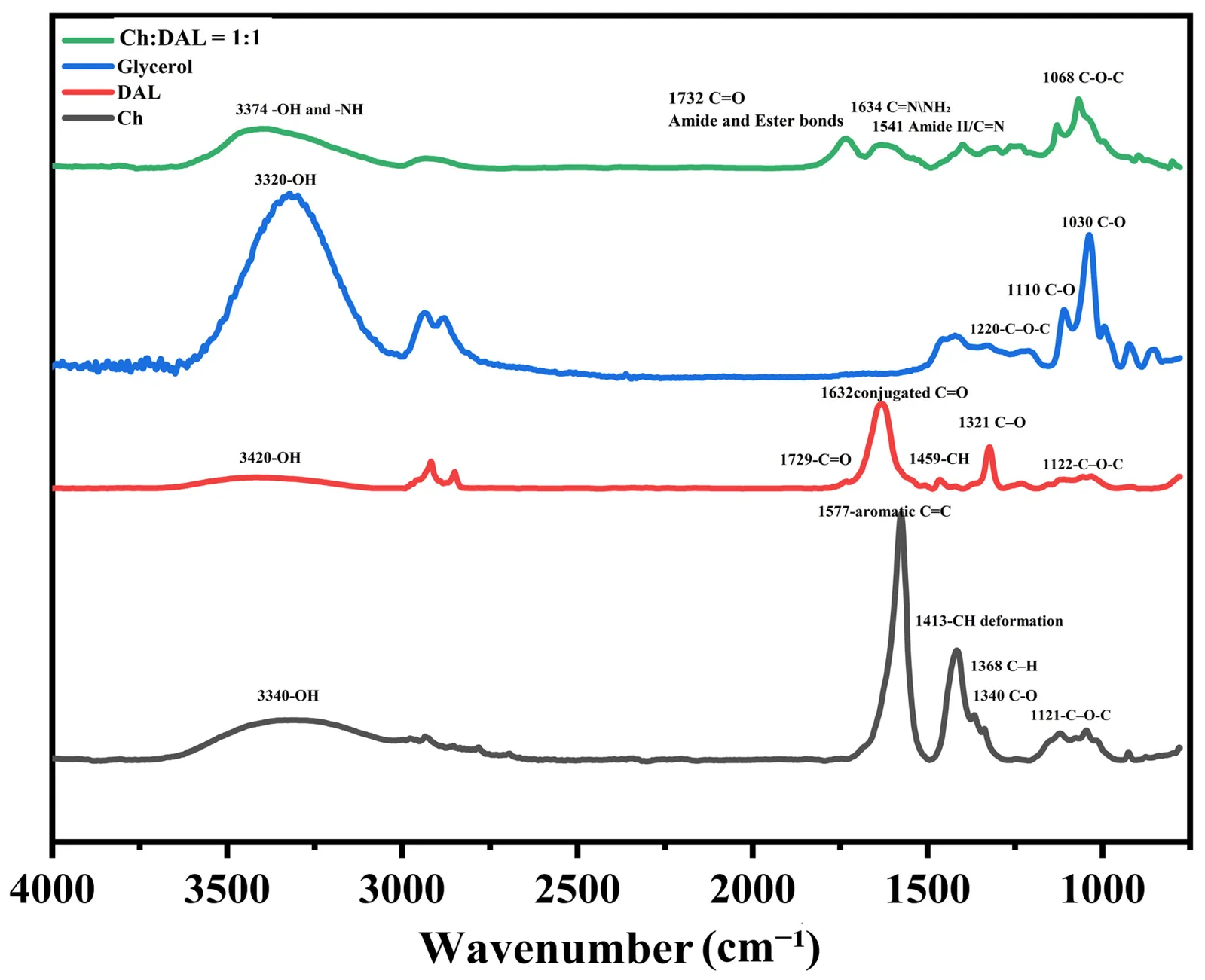
FTIR spectra of chitosan (Ch), dialdehyde lignin (DAL), glycerol, and the Ch-DAL bioplastic prepared at a Ch:DAL ratio of 1:1. The band near 1634 cm^−1^ may contain overlapping contributions from imine (C=N) stretching and the chitosan amide I region, whereas the band near 1541 cm^−1^ is assigned primarily to the amide II region. These spectral features support, but do not directly quantify, the proposed formation of imine linkages in the Ch-DAL network.

**Figure 4:**
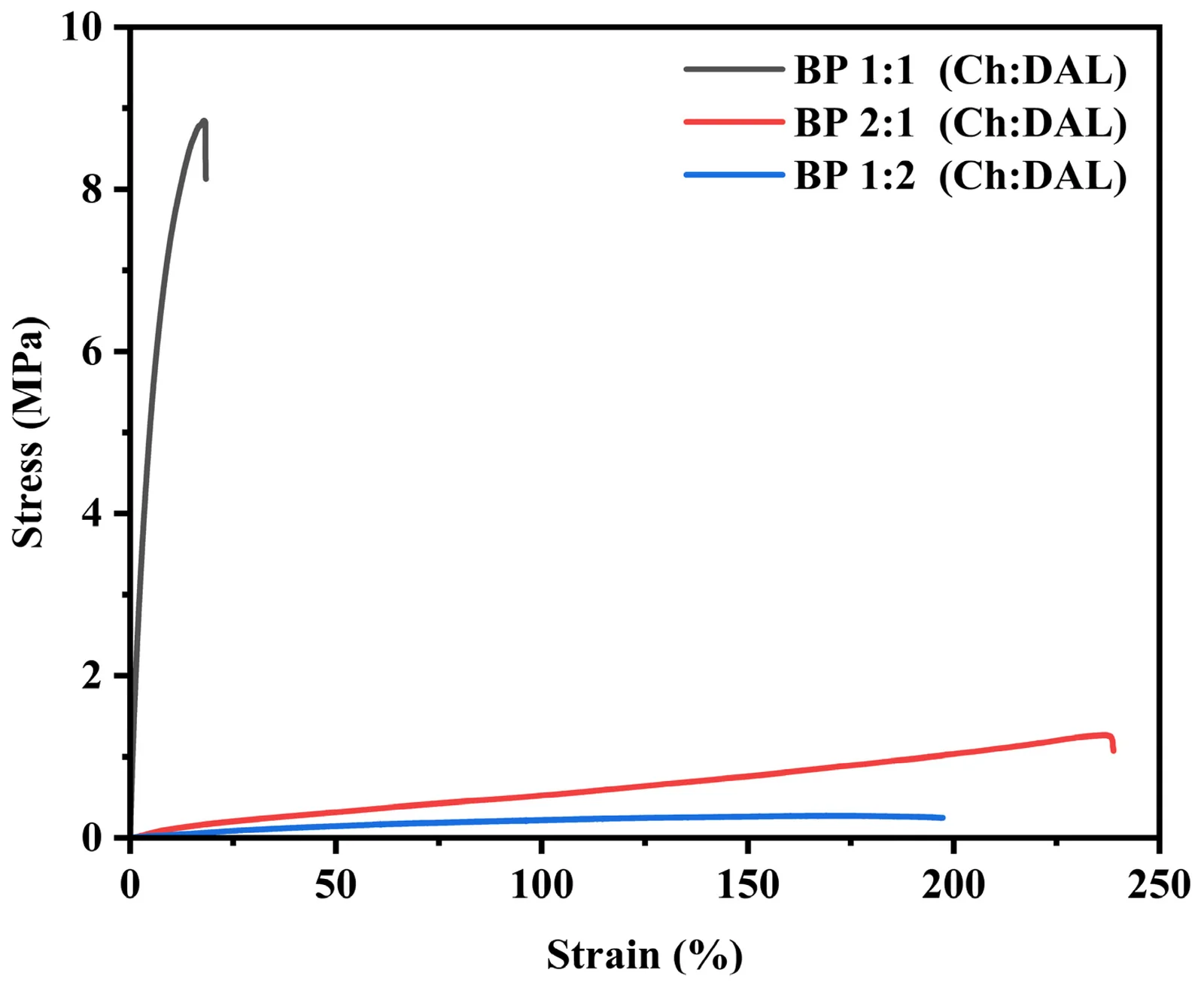
Stress–strain behavior of Ch-DAL bioplastics with different chitosan-to-dialdehyde lignin (Ch:DAL) ratios. One representative curve is shown for each formulation from three independently prepared and tested specimens (n = 3).

**Figure 5:**
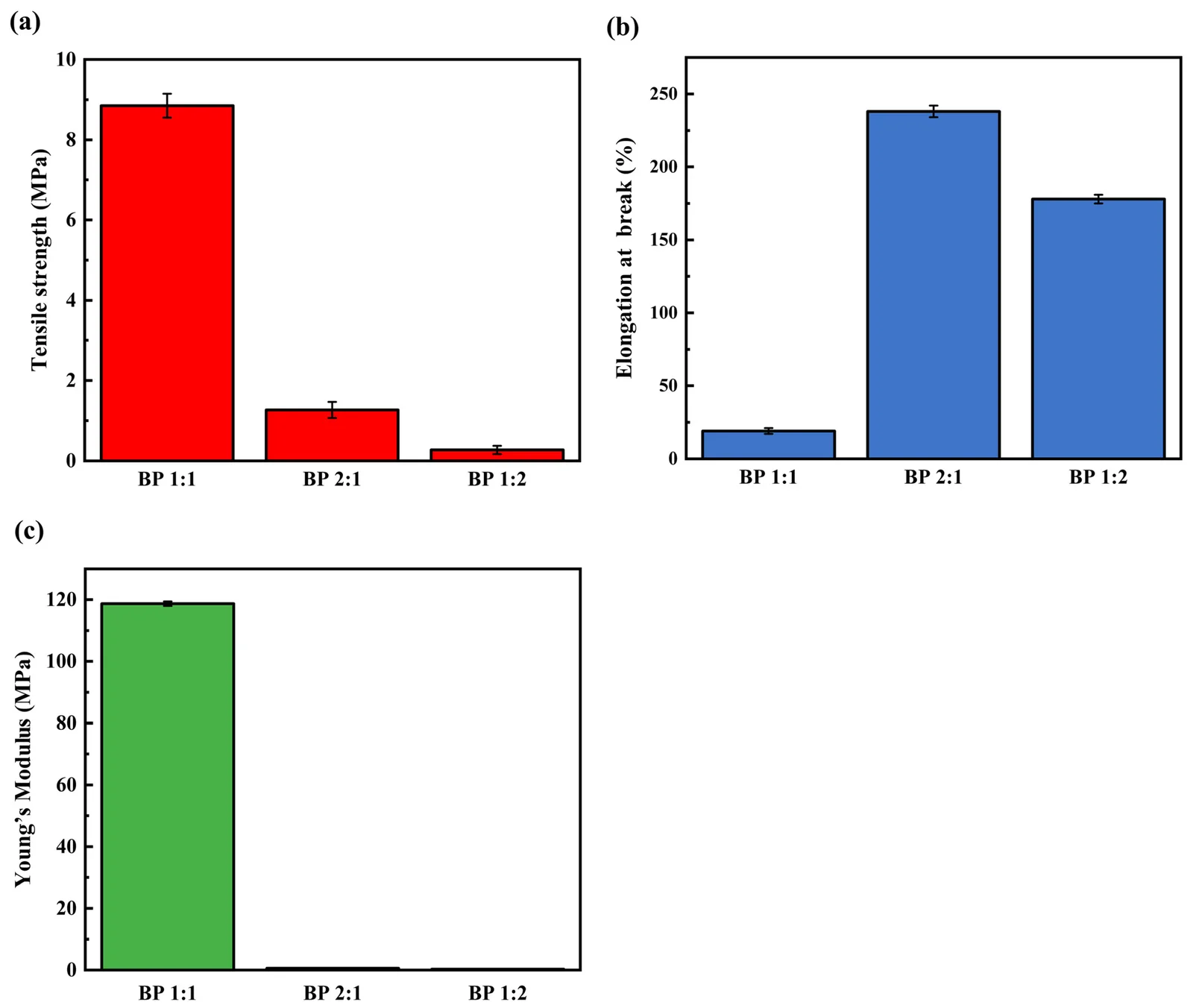
Mechanical properties of Ch-DAL bioplastics with different Ch:DAL ratios: (**a**) tensile strength, (**b**) elongation at break, and (**c**) Young’s modulus. Bars represent the mean, and error bars represent the standard deviation (SD) of three independently prepared specimens for each formulation (n = 3).

**Figure 6:**
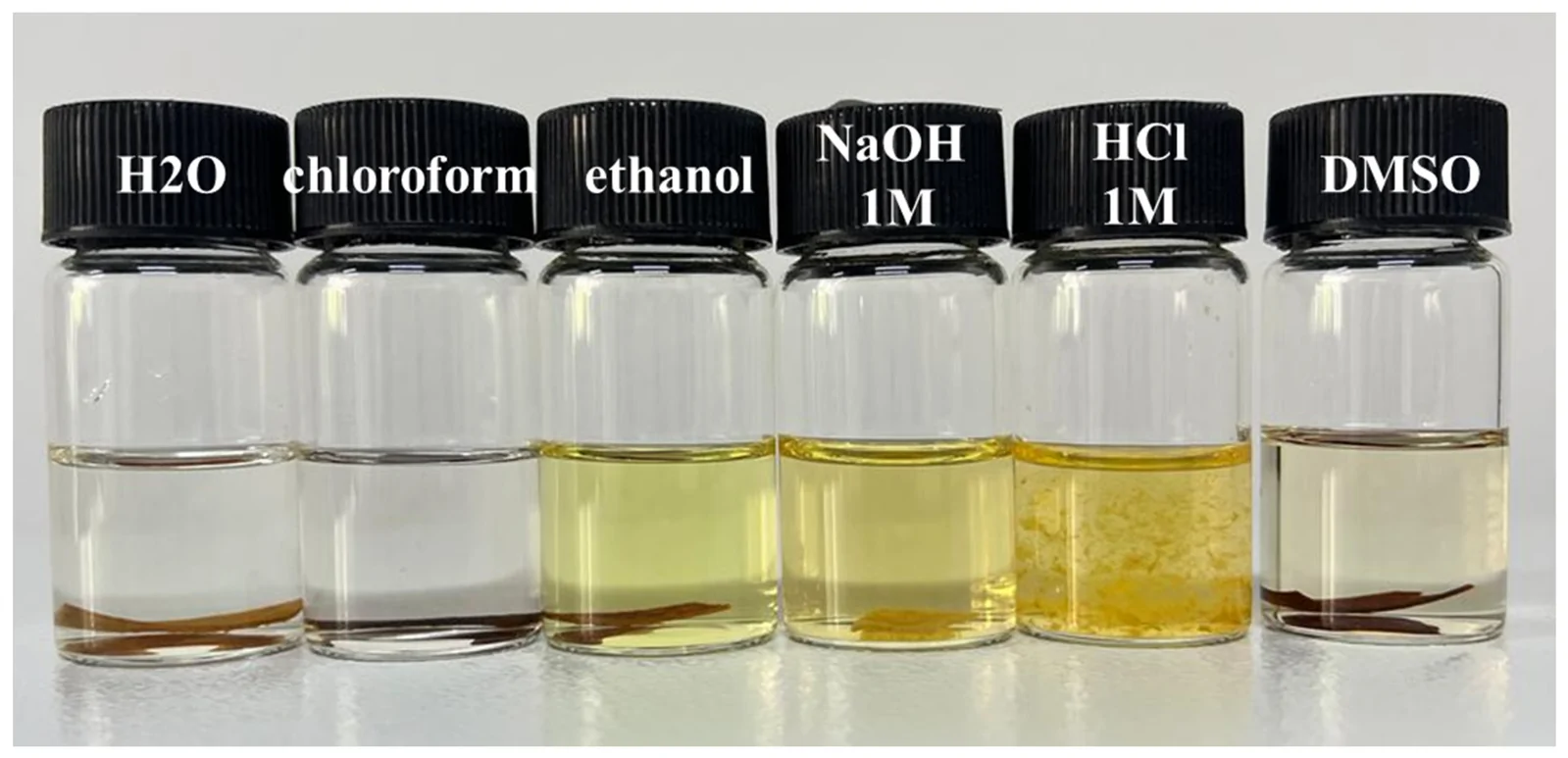
Visual appearance of Ch-DAL bioplastic (Ch:DAL = 1:1) after immersion in H_2_O, chloroform, ethanol, 1 M NaOH, 1 M HCl, and DMSO for 24 h at room temperature (approximately 25 °C).

**Figure 7:**
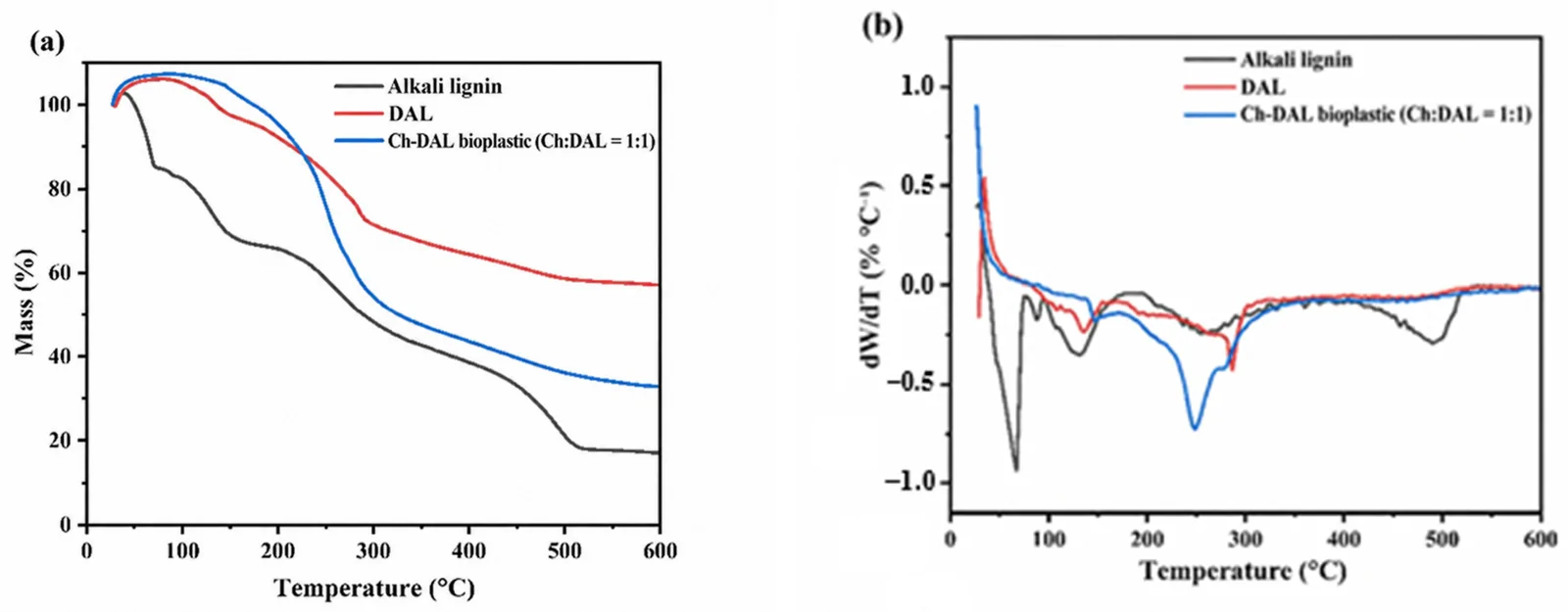
Thermogravimetric (TGA) (**a**) and derivative thermogravimetric (DTG) (**b**) curves of alkali lignin, dialdehyde lignin (DAL), and the Ch–DAL bioplastic film (Ch:DAL = 1:1).

**Figure 8:**
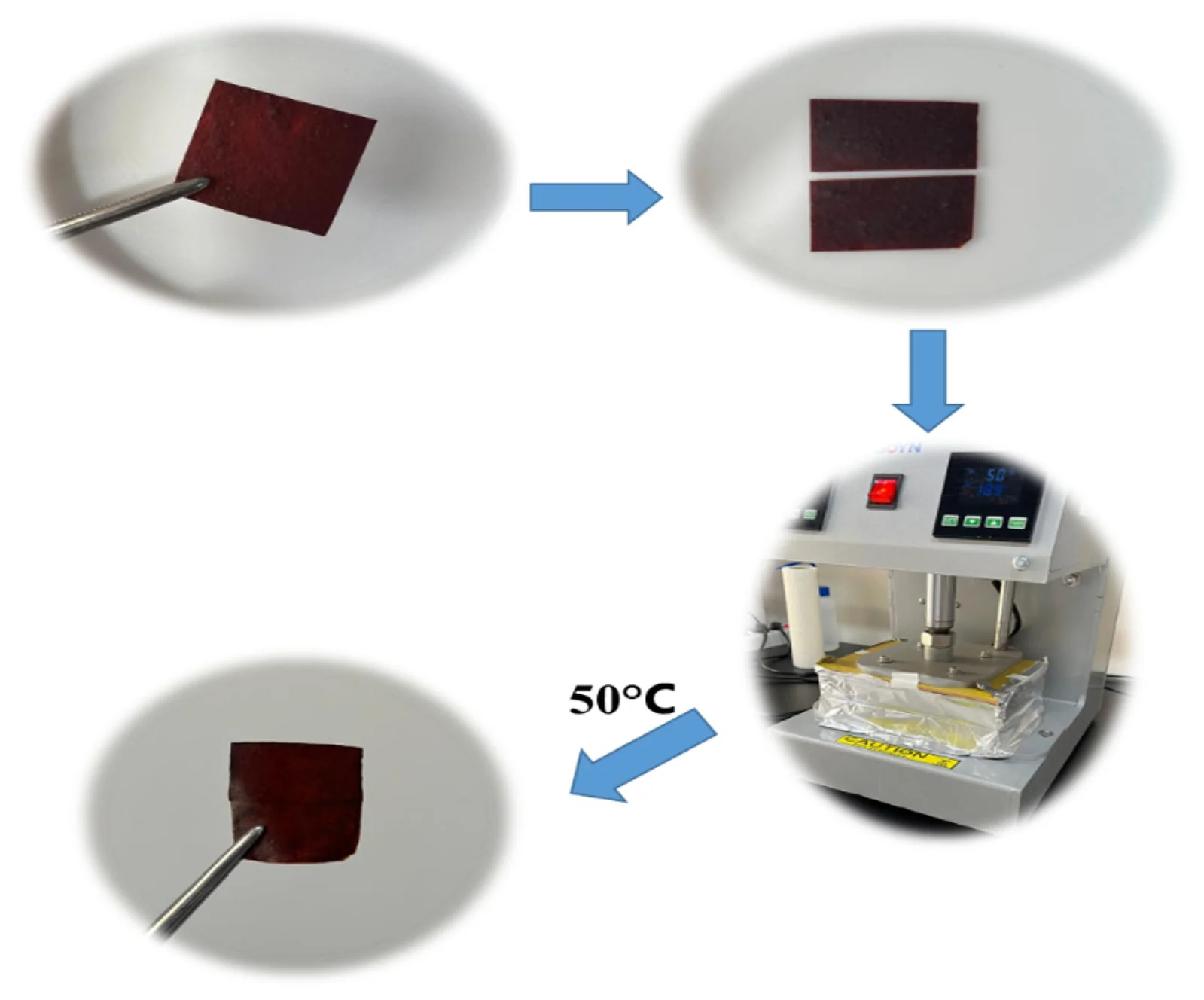
Macroscopic thermally assisted repair of the Ch-DAL bioplastic following cutting and hot pressing at 50 °C for 10 min.

**Figure 9:**
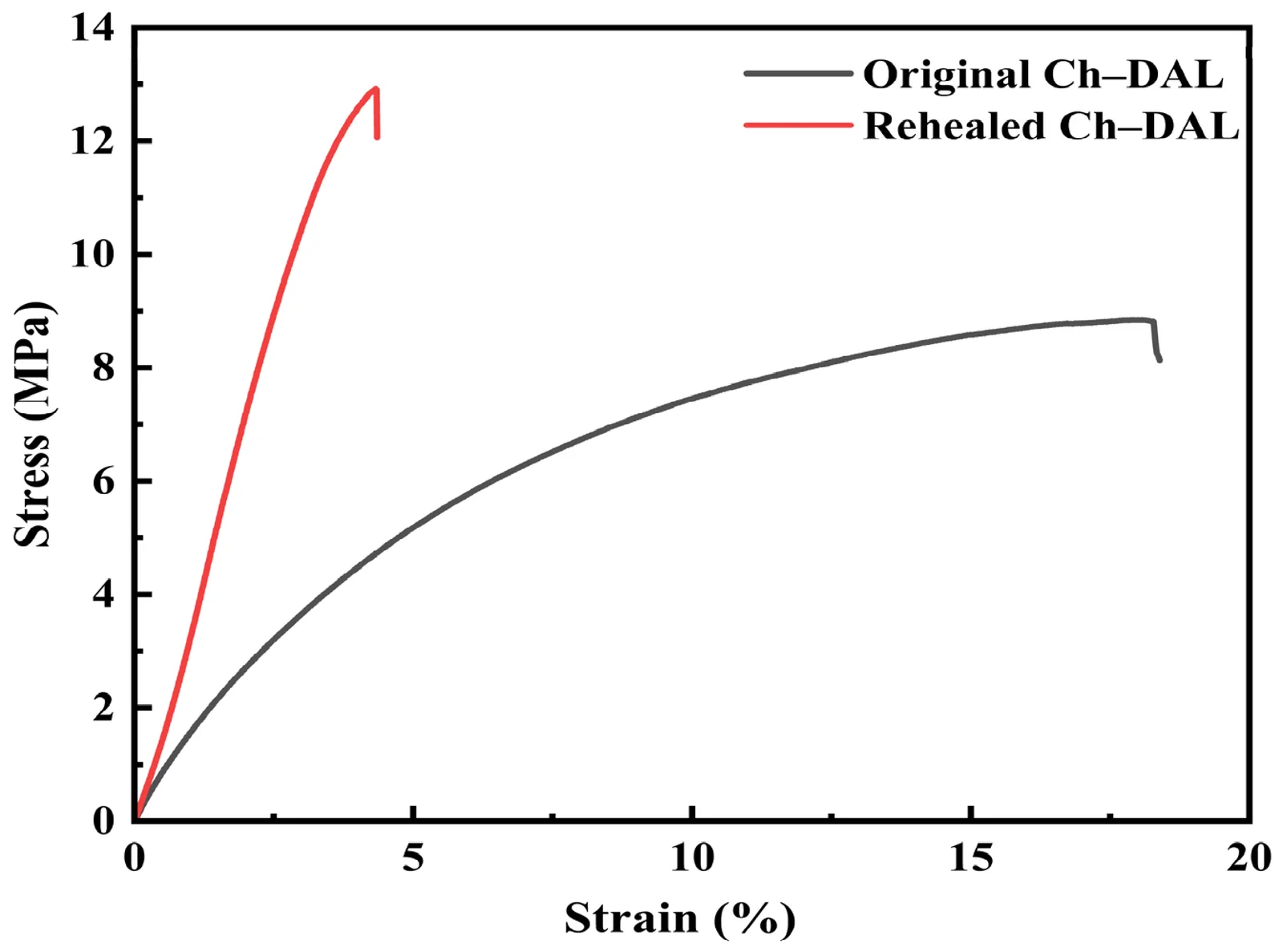
Stress–strain behavior of the original and thermally repaired Ch-DAL bioplastic films. One representative curve is shown for each condition from three independently prepared and tested specimens (n = 3).

**Figure 10:**
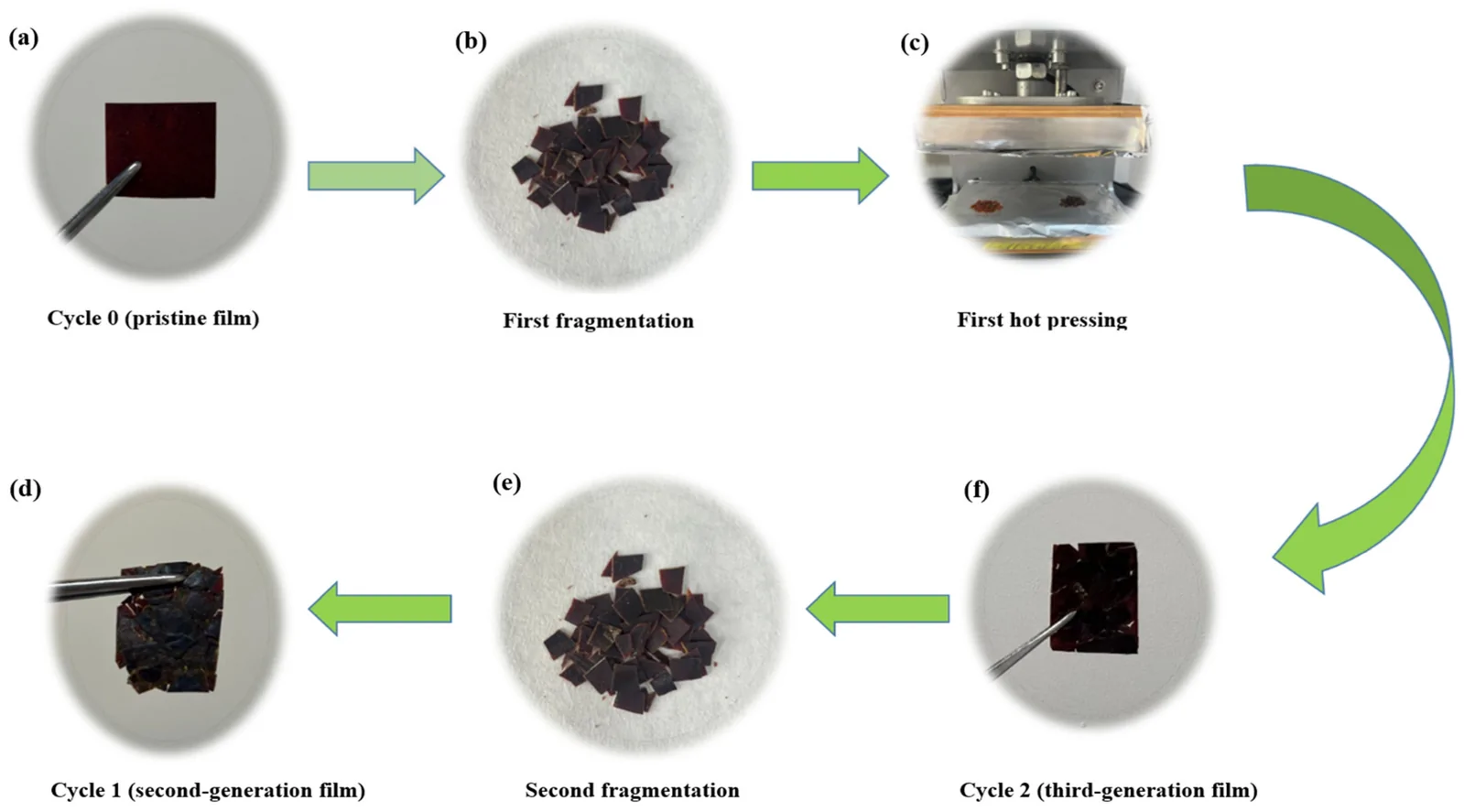
Thermo-mechanical reprocessing of the 1:1 Ch-DAL bioplastic: (**a**) cycle 0 (pristine film), (**b**) first fragmentation, (**c**) first hot-pressing step between Teflon sheets at 50 °C for 10 min using a BOYN hot press, (**d**) cycle 1 (second-generation film), (**e**) second fragmentation, and (**f**) cycle 2 (third-generation film). The applied pressure was not quantitatively recorded.

**Figure 11:**
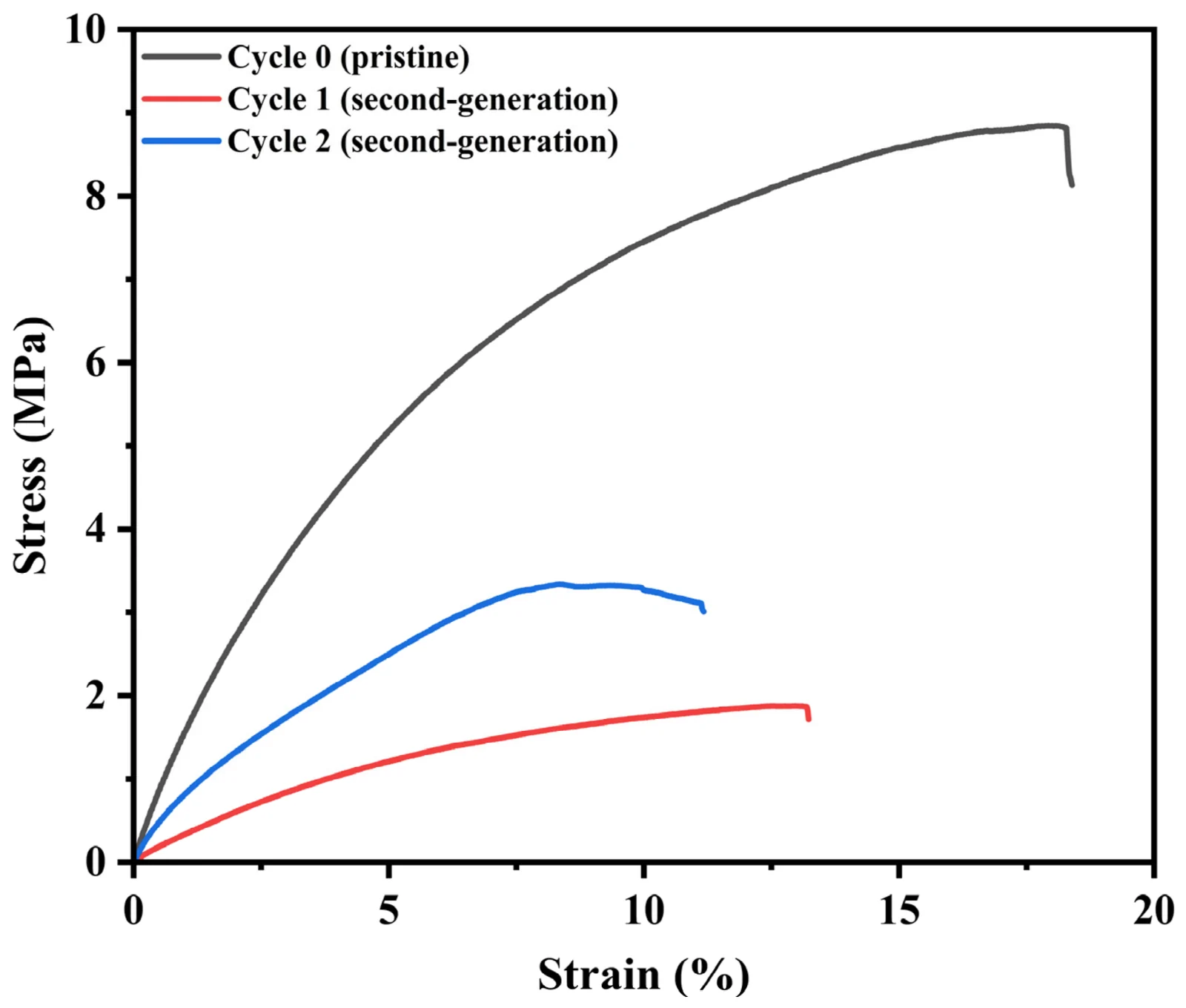
Stress–strain behavior of the 1:1 Ch-DAL bioplastic during successive thermo-mechanical reprocessing cycles: cycle 0 (pristine), cycle 1 (second-generation), and cycle 2 (third-generation) films. One representative curve is shown for each processing condition from three independently prepared and tested specimens (n = 3).

**Figure 12:**
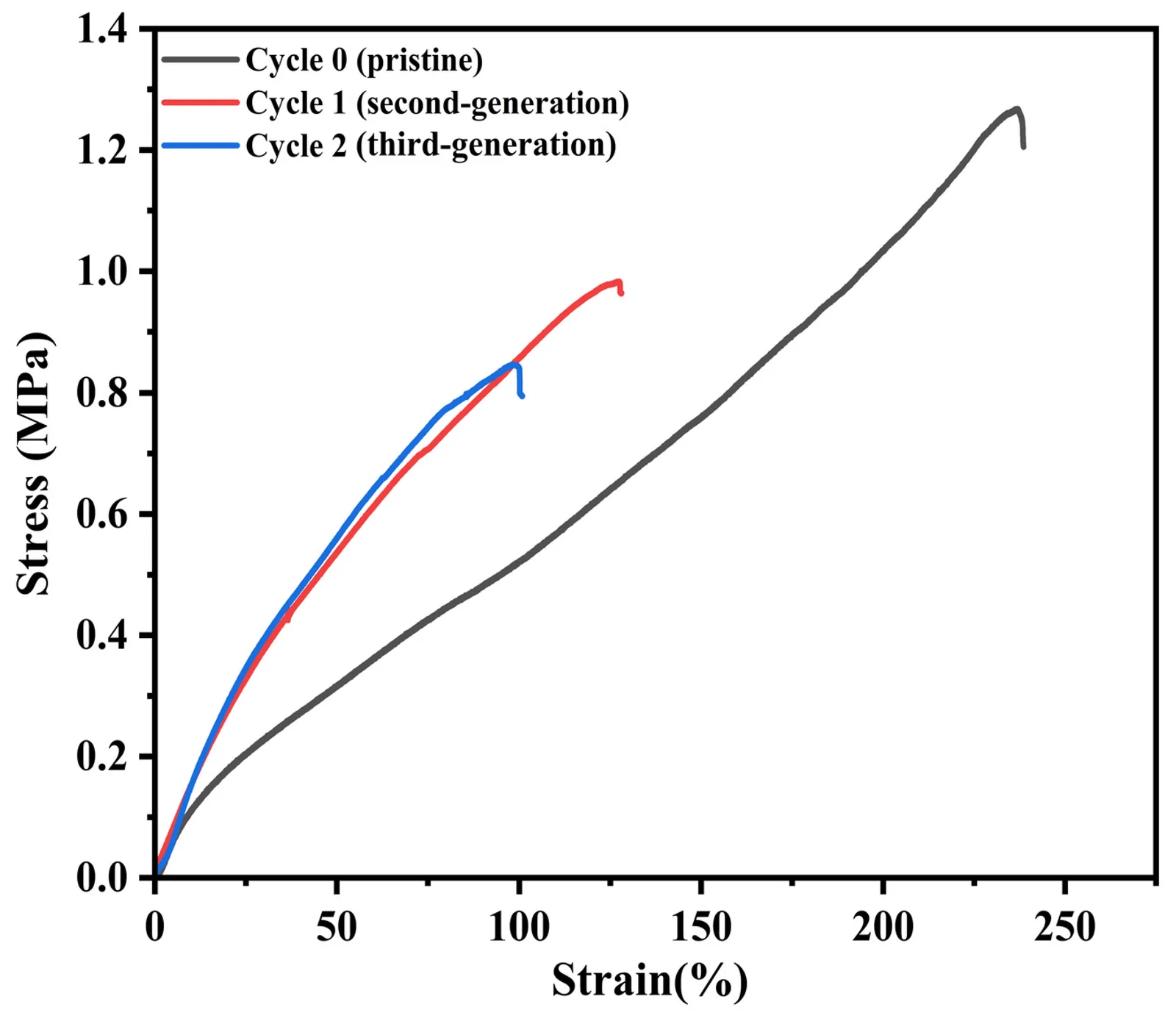
Stress–strain behavior of the 2:1 Ch-DAL bioplastic during successive thermo-mechanical reprocessing cycles: cycle 0 (pristine), cycle 1 (second-generation), and cycle 2 (third-generation) films. One representative curve is shown for each processing condition from three independently prepared and tested specimens (n = 3).

**Table 1:** Composition of the Ch-DAL film-forming formulations.

Formulation	Ch:DAL Mass Ratio	Chitosan (g)	DAL (g)	3% (w/v) Tartaric Acid Solution (mL)	Glycerol (mL)	Glycerol Relative to Ch-DAL Mass (wt%)	Chitosan Concentration (% w/v)	Total Biopolymer Concentration (% w/v)	Nominal Total Formulation Content (% w/v)
Ch-DAL 1:1	1:1	0.70	0.70	35.0	0.20	17.9	2.00	4.00	7.71
Ch-DAL 2:1	2:1	0.47	0.23	17.5	0.10	17.9	2.69	4.00	7.71
Ch-DAL 1:2	1:2	0.23	0.47	17.5	0.10	17.9	1.31	4.00	7.71

**Table 2:** Mechanical properties of Ch-DAL bioplastics prepared at different chitosan-to-dialdehyde lignin (Ch:DAL) ratios.

Ch:DAL Ratio	Tensile Strength (MPa)	Elongation at Break (%)	Young’s Modulus (MPa)
1:1	8.85 ± 0.09	19 ± 0.1	118.7 ± 0.7
2:1	1.27 ± 0.05	238 ± 0.1	0.570 ± 0.002
1:2	0.27 ± 0.07	178 ± 0.2	0.2880 ± 0.0005

**Table 3:** Mechanical properties of the original and thermally repaired Ch-DAL bioplastic films.

Sample	Tensile Strength (MPa)	Elongation at Break (%)	Young’s Modulus (MPa)
Original Ch-DAL bioplastic	8.85 ± 0.09	19 ± 0.1	118.7 ± 0.7
Thermally repaired Ch-DAL bioplastic	12.9 ± 0.11	4.3 ± 0.05	316.9 ± 1.6

**Table 4:** Mechanical properties of pristine and thermo-mechanically reprocessed 1:1 Ch-DAL bioplastics.

Reprocessing Stage	Tensile Strength (MPa)	Elongation at Break (%)	Young’s Modulus (MPa)
Cycle 0 (pristine)	8.85 ± 0.09	19 ± 0.1	118.7 ± 0.7
Cycle 1 (second-generation)	1.87 ± 0.2	13 ± 0.04	28.734 ± 0.123 MPa
Cycle 2 (third-generation)	3.10 ± 0.05	11 ± 0.03	75.847 ± 0.826 MPa

**Table 5:** Mechanical properties of pristine and thermo-mechanically reprocessed 2:1 Ch-DAL bioplastics.

Reprocessing Stage	Tensile Strength (MPa)	Elongation at Break (%)	Young’s Modulus (MPa)
Cycle 0 (pristine)	1.27 ± 0.04	238 ± 0.1	0.570 ± 0.002
Cycle 1 (second-generation)	0.98 ± 0.02	128 ± 0.1	1.223 ± 0.002
Cycle 2 (third-generation)	0.84 ± 0.05	100 ± 0.05	1.470 ± 0.002

## Data Availability

The data supporting the findings of this study, including the raw mechanical-testing and FTIR data, are available from the corresponding authors upon reasonable request.

## Supplementary Materials

The following supporting information can be downloaded at: https://www.mdpi.com/article/10.3390/ma19194064/s1, Figure S1: Schematic illustration of tensile-specimen preparation and thermally assisted repair.
