# Supplementary material for: Dynamic Schiff Base Chitosan–Lignin Bioplastics with Thermally Assisted Repair and a Reprocessable Network
Source: Materials (Basel). 2026 Sep 23;19(19):4064. doi: 10.3390/ma19194064 (PMC13665790; doi:10.3390/ma19194064)
Supplement: Supplementary file 1 [file materials-19-04064-s001.zip › materials-4551504-Figure S1.pdf]

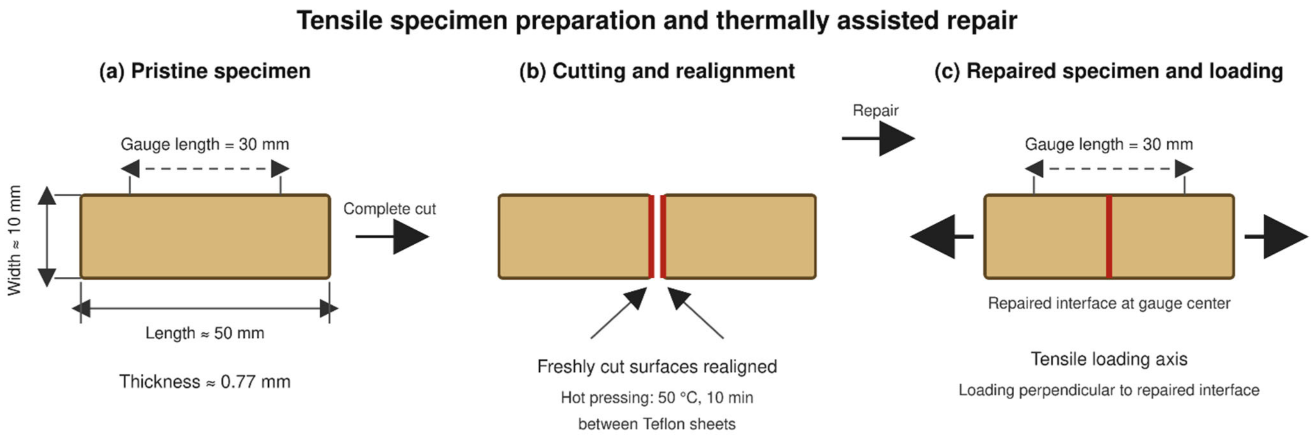

Figure S1. Schematic illustration of tensile-specimen preparation and thermally assisted repair. (a) Pristine rectangular specimen with approximate dimensions of 50 mm × 10 mm × 0.77 mm and an initial gauge length of 30 mm. (b) Complete transverse cutting and realignment of the freshly cut surfaces before hot pressing between Teflon sheets at 50 °C for 10 min. (c) Thermally repaired specimen with the repaired interface positioned approximately at the center of the gauge region. Tensile loading was applied perpendicular to the repaired interface. The applied pressure was not instrumentally controlled or quantitatively recorded.
